# Impact of long-stay beds on the performance of a tertiary hospital in emergencies

**DOI:** 10.1590/S0034-8910.2015049006078

**Published:** 2015-11-16

**Authors:** Antonio Pazin, Edna de Almeida, Leni Peres Cirilo, Frederica Montanari Lourençato, Lisandra Maria Baptista, José Paulo Pintyá, Ronaldo Dias Capeli, Sonia Maria Pirani Felix da Silva, Claudia Maria Wolf, Marcelo Marcos Dinardi, Sandro Scarpelini, Maria Cecília Damasceno

**Affiliations:** I Divisão de Emergências Clínicas. Departamento de Clínica Médica. Faculdade de Medicina de Ribeirão Preto. Universidade de São Paulo. Ribeirão Preto, SP, Brasil.; II Unidade de Emergência. Hospital das Clínicas. Faculdade de Medicina de Ribeirão Preto. Universidade de São Paulo. Ribeirão Preto, SP, Brasil; III Departamento de Atenção à Saúde. Unidade de Emergência. Hospital das Clínicas. Faculdade de Medicina de Ribeirão Preto. Universidade de São Paulo. Ribeirão Preto, SP, Brasil; IV Departamento Regional de Saúde XIII. Secretaria de Saúde do Estado de São Paulo. Ribeirão Preto, SP, Brasil; V Divisão de Cirurgia do Trauma. Departamento de Cirurgia e Anatomia. Faculdade de Medicina de Ribeirão Preto. Universidade de São Paulo. Ribeirão Preto, SP, Brasil; VI Departamento Clínico-Cirúrgico. Faculdade de Medicina do ABC. São Paulo, SP, Brasil

**Keywords:** Bed Occupancy, Hospital Bed Capacity, Length of Stay, Long-Term Care, Tertiary Healthcare, Emergency Medical Services, Charlson Comorbidity Index

## Abstract

**OBJECTIVE:**

To assess the impact of implementing long-stay beds for patients of low complexity and high dependency in small hospitals on the performance of an emergency referral tertiary hospital.

**METHODS:**

For this longitudinal study, we identified hospitals in three municipalities of a regional department of health covered by tertiary care that supplied 10 long-stay beds each. Patients were transferred to hospitals in those municipalities based on a specific protocol. The outcome of transferred patients was obtained by daily monitoring. Confounding factors were adjusted by Cox logistic and semiparametric regression.

**RESULTS:**

Between September 1, 2013 and September 30, 2014, 97 patients were transferred, 72.1% male, with a mean age of 60.5 years (SD = 1.9), for which 108 transfers were performed. Of these patients, 41.7% died, 33.3% were discharged, 15.7% returned to tertiary care, and only 9.3% tertiary remained hospitalized until the end of the analysis period. We estimated the Charlson comorbidity index – 0 (n = 28 [25.9%]), 1 (n = 31 [56.5%]) and ≥ 2 (n = 19 [17.5%]) – the only variable that increased the chance of death or return to the tertiary hospital (Odds Ratio = 2.4; 95%CI 1.3;4.4). The length of stay in long-stay beds was 4,253 patient days, which would represent 607 patients at the tertiary hospital, considering the average hospital stay of seven days. The tertiary hospital increased the number of patients treated in 50.0% for Intensive Care, 66.0% for Neurology and 9.3% in total. Patients stayed in long-stay beds mainly in the first 30 (50.0%) and 60 (75.0%) days.

**CONCLUSIONS:**

Implementing long-stay beds increased the number of patients treated in tertiary care, both in general and in system bottleneck areas such as Neurology and Intensive Care. The Charlson index of comorbidity is associated with the chance of patient death or return to tertiary care, even when adjusted for possible confounding factors.

## INTRODUCTION

One of the biggest problems of the Brazilian Unified Health System (SUS) is transferring chronic patients with high dependency (needing aid for basic life functions), but which are no longer of high complexity (do not require tertiary medical care, diagnostic or therapeutic resources).^4^ ^,^ ^7^ This implies a prolonged stay of these patients in emergency rooms and tertiary inpatient hospital beds, causing internal and external friction,^19^ ^,^ ^20^ work overload for the nursing staff and high hospital costs.^3^ ^,^ ^8^ ^,^ ^10^

Installing long-stay beds is a possible solution to this issue.^5^ They are meant to be placed in small hospitals, which face enormous economic difficulties and have low occupancy rates.^10^ In addition, these institutions have difficulty in dealing with high dependency patients with special needs as dependents of noninvasive ventilation. The unpreparedness of these institutions to deal with high dependency causes a great number of acute exacerbations of chronic conditions, with high rates of counter-referral to emergency rooms.^1^ ^,^ ^2^ ^,^ ^16^ To make installation possible, different inpatient *per diem* rates and an initial aid for qualifying costs are planned.^a^

This study aims to assess the impact of implementing long-stay beds for patients of low complexity and high dependency in small hospitals on the performance of an emergency referral tertiary hospital.

## METHODS

The study involved the tertiary-care emergency hospital and partner hospitals of the 13^th^ regional department of health (DRS XIII). The emergency department of Hospital das Clínicas of the Faculdade de Medicina de Ribeirão Preto of the Universidade de São Paulo (HCFMRP-USP) has 169 beds (30.0% intensive care), with a referral emergency room, which serves as tertiary referral for emergencies of the DRS XIII and four other departments. It has high-complexity diagnostic and treatment resources and is the only referral facility for some clinical conditions within a radius of 300 kilometers in the northeastern state of Sao Paulo, in Brazil.^1^ ^,^ ^14^

**Figure 1:**
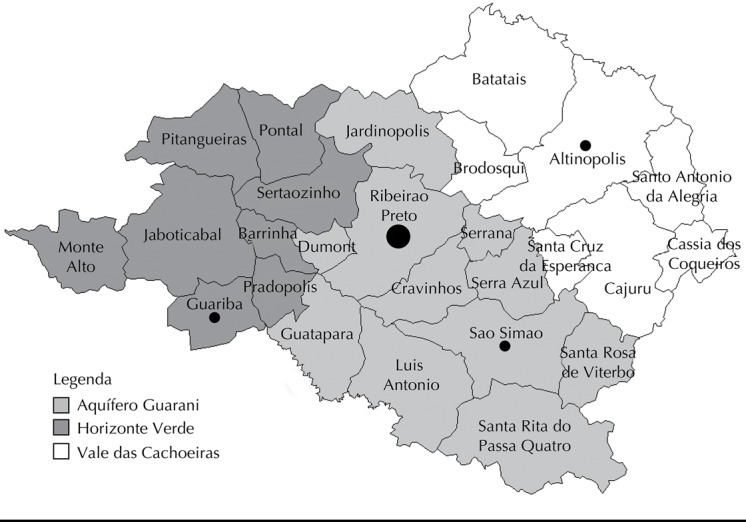
Municipalities in the 13th regional department of health of the state of Sao Paulo, according to district. The larger sphere marks the hub municipality and the smaller spheres, the project partner municipalities, showing the strategic location of each district.

The coordination of the emergency department, the DRS XIII, the Ribeirao Preto City Hall and the regional emergency medical ambulance service (SAMU) identified three hospitals to establish partnerships in the municipalities of Sao Simao, Guariba and Altinopolis (Figure 1). Technical visits were carried out and long-stay patient referral protocols were defined, registering the capabilities and responsibilities of each institution.

Partner hospitals were selected based on the profile required by Ordinance 2809 of December 7, 2012, and on the willingness to take the risk of carrying out the project with their own resources until receiving those offered by the state of Sao Paulo. The involvement in the project did not occur at the same time for all hospitals, since the time they needed to prepare for receiving patients varied among them, with a three-month interval between the first and the last one. These hospitals had different initial conditions to receive patients. One of them was already prepared for more severe patients, while the others had to be qualified. The strategic position of these municipalities was also considered (Figure 1).

An agreement was signed between participants so that the process was beneficial for all parties (“win-win”). The tertiary hospital would free beds to receive more high-complexity patients. Partner hospitals would have the prospect of financial gain, because for each patient day they would be given an incentive of BRL 300.00 (three hundred reais).^b^ In addition, they would be connected to the tertiary hospital and receive qualification from its employees.

After in locu visits, a transfer protocol was established based on partners’ physical and staff conditions. The transfer process started with the agreement from patients and their family members, and provided an on-site visit of a family member to the partner hospital to evaluate the conditions and the guarantee of transport assistance by the city halls of the municipalities in which the patients resided so that family members could continue to visit them after the transfer. It was clarified that, if there was any need to return the patient to a tertiary hospital, it would be done regardless of the Medical Regulation.

After the agreement from the patient and family members, a bed request was made to the partner municipality, chosen by proximity from their home, bed availability or for specific hospital conditions matching the problem presented by the patient. No patient was transferred without the mutual agreement of the health teams of the institutions (tertiary and partner) and of the patient or family member. If the partner city agreed to the transfer, the patient was referred with medical, nursing, physical therapy, nutritional, psychological and social assistance reports. These reports detailed patient needs so that treatment could be continued in the partner hospital.

Five beds were initially established in each partner hospital. As financial resources were still not available at this stage (September 2013 to March 2014), each institution involved in the project contributed in some way, e.g., with the supply of adult diapers or medicines. The regional SAMU were all scheduled and occurred from Monday to Friday during business hours. Staff of the partner hospitals was trained by the referral hospital on care that limited transfers such as tracheostomy management, use of BiPAP and preparation of special diets.

Starting in April 2014, the project was sanctioned and financially supported by the Department of Health of the State of Sao Paulo. In this stage, the number of beds in each partner was raised to 10, totaling 30 long-stay beds for DRS XIII. Other hospitals in the hub municipality began to transfer patients following the guidelines created. Payment was made by DRS XIII with resources from the Department of the State of Sao Paulo, according to the production presented. If a partner kept its 10 beds occupied the whole month, their revenue would correspond to three to four times its total monthly revenue.

The tertiary hospital and the DRS XIII monitored all patients hospitalized in partners daily. Patients ceased to be monitored only when they left the partner hospital (by death or discharge), making these hospitals an extension of the tertiary one.

Categorical variables were expressed as percentages and quantitative variables as mean and standard deviation or median and interquartile range, according to their distribution. Fisher’s test and Chi-square test were used to compare categorical variables. To compare continuous variables, the parametric Student’s t-test and the analysis of variance (ANOVA) or nonparametric equivalents were used. Survival analysis was used to evaluate the length of stay of referred patients in each partner hospital. In these cases, discharge, death or transfer were considered outcomes.

Cox semiparametric regression and logistic regression were used for multivariate analysis, considering death or transfer as outcomes. The actual period the patient stayed in the partners was used as an outcome (censoring by discharge, death, transfer or being hospitalized until September 30, 2014). Sensitivity analysis was performed to evaluate death as an outcome. For both strategies, incremental models (forward) were built until the final model adjusted for age, gender, partner municipality, and Charlson index. The Charlson index of comorbidity was estimated based on the international code of diseases ICD-10.^12^ For all tests, statistical significance was considered when p < 0.05. For data analysis and graph building, the software Stata version 10, Microsoft Excel^®^ and ArcGIS version 9 were used.

The project was approved by the Research Ethics Commit- tee of HCFMRP-USP (CAE 30686214.2.0000.5440). It was exempted of the need for an informed consent form for patients because it is an observational study involving administrative data.

## RESULTS

Between September 1, 2013, and September 30, 2014, we included 97 patients (72.1% male), with a mean age of 60.5 years (SD = 1.9), for which 108 transfers were performed. Table 1 describes the distribution according to partner hospital, unit of origin and outcome in the partner institution. The transfers occurred mainly for patients with cardiovascular problems and degenerative diseases (Clinical Group), stroke and other neurological disorders (Neurological Group), and trauma (Surgical Group), with the aim of completion of treatment (Table 1).

**Table 1:** Distribution of transfers carried out according to municipality, unit of origin type and outcome in the long-stay hospital.

	Discharge	Death	Hospitalized	Return	Total	%
Altinopolis (25.9%)						
Clinical Group	3	2	1	0	6	5.6
Neurological Group	6	4	0	3	13	12.0
Surgical Group	5	3	0	1	9	8.3
Guariba (56.5%)						
Clinical Group	3	9	1	4	17	15.7
Neurological Group	6	14	2	3	25	23.1
Surgery	11	4	2	2	19	17.6
Sao Simao (17.6%)						
Clinical Group	1	1	1	1	4	3.7
Neurological Group	0	7	2	2	11	10.2
Surgical Group	1	1	1	1	4	3.7
Total	36	45	10	17	108	
%	33.3	41.7	9.3	15.7		

Note: Neurological transfers corresponded to 45.4% of transfers, while those for clinical and surgical reasons corresponded to 25.0% and 29.6%, respectively.

We estimated the Charlson index of comorbidity for patients referred to partner hospitals (Table 2). With respect to the outcome, we observed an index ≥ 2 in 5.0% of patients discharged, 20.0% of those hospitalized, 35.0% of those who had to return to the institution of origin and 40.0% of those who died.

**Table 2:** Charlson index according to partner municipality.*

	0		1		≥ 2		Total	
Partner	n	%	n	%	n	%	n	%
Altinopolis	11	23.4	12	36.3	5	17.8	28	25.9
Guariba	30	63.8	17	51.5	14	50.0	61	50.5
Sao Simao	6	12.7	4	12.1	9	32.1	19	17.6
Total	47		33		28		108	100

* Percentages correspond to table columns.

**Figure 2:**
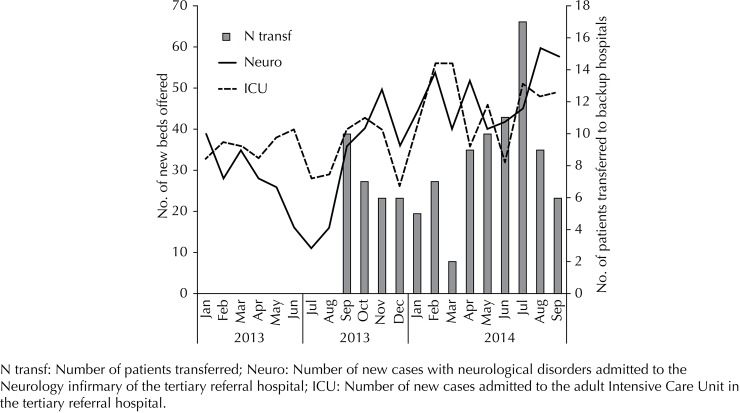
Number of new beds offered in Intensive Therapy and Neurology (Lines - Y Axis to the left) by the tertiary institution according to the number of patients transferred to long-stay hospitals (Columns - Y Axis to the right) *versus* time (X Axis). The unfilled arrow marks the beginning of Stage 1 of the project and the filled arrow marks the beginning of Stage 2.

Figure 2 illustrates the impact of long-stay beds on the number of new beds for Intensive Care and Neurology by month. Regarding beds in general, these and the other clinics benefited totaled a 9.3% increase in new beds offered. We observed this total number of discharges after an increase from 15 to 30 long-stay beds.

The total stay in the tertiary hospital and long-stay beds was 9,134 patient days. Of these, 4,881 were of the tertiary hospital (53.5%) 4,253 of the long-stay beds (46.5%). Considering the mean length of stay of seven days in the tertiary hospital, the length of stay in partner hospitals would allow approximately 607 new patients being treated.

**Figure 3:**
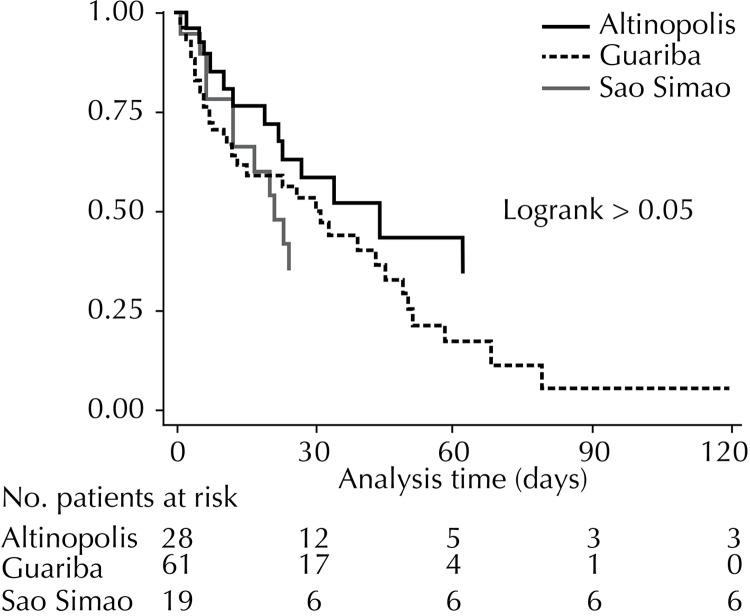
Kaplan-Meier graph for the length of stay of patients in each institution.

There was no significant difference among partner hospitals, both in univariate analysis and multivariate analysis by logistic regression or Cox semiparametric regression (Figure 3). About 50.0% of the patients were discharged, died or were transferred within the first 30 days. The Charlson index was the only variable that significantly increased the chance of death or return to the tertiary hospital (Odds Ratio – 2.4; 95%CI 1.3;4.4), but there was no significance regarding the length of stay in the Cox regression (Odds Ratio – 1.2; 95%CI 0.8;1.7) (Figure 3).

The municipalities were similar (p > 0.05) regarding percentage of deaths – Altinopolis (9; 32.1%), Guariba (27; 44.2%), and Sao Simao (9; 47.3%). Death were more frequent in the Neurological Group (25; 51%), followed by the Clinical Group (12; 44.4%) and the Surgical Group (8; 25.0%). According to the codes of ICD-10, the main causes of death were: Diseases of the Circulatory System (20; 45.5%); Trauma (7; 15.9%), Diseases of the Respiratory System (7; 15.9%), Neoplasms (4; 9.1%), and Others (6; 13.6%). Examining the most frequent ICD-10 codes, we observed that those resulting from cerebrovascular diseases accounted for 29.5% of cases, of which 60.0% were admitted in Guariba. Considering only the death as an outcome in Cox regression analysis, none of the variables included in the model was significant. In multivariate logistic regression analysis, the Charlson index remained a significant variable – OR = 1.7 (1.0;2.9) – and there was greater chance of death in patients referred to Guariba – OR = 1.8 (1.0;2.9).

## DISCUSSION

Implementing long-stay beds increased significantly the number of beds offered in tertiary care in system bottleneck areas as Neurology and Intensive Care. After the expansion of the project to 30 beds, we observed a trend of increase in the general offer of beds in the tertiary hospital. The degree of comorbidity, measured by the Charlson index, was associated with the chance of patient death or return to tertiary care, even when adjusted for possible confounding factors. Patients stayed in partner hospitals mainly in the first 30-60 days.

Full management of health by the municipalities has created the understanding that each municipality should be responsible for providing such support for long-stay patients, transferring them to primary instances of SUS located in the municipalities where patients dwell. Such transfers are desirable, especially for the benefit of maintaining family support. However, most municipalities use the system of service purchase of public or charity hospitals, i.e., many municipalities have just one long-stay institution or none at all, which hampers transferring.

A possible strategy would be the dehospitalization of these patients to their homes, adding the strategic support of *Saúde da Família* (Family Health) teams and *Serviço de Apoio Domiciliar* (Home Care Service). Although this strategy is inviting, it is hindered by various problems: social (carers are obliged to leave their productive activities, with financial repercussions for the household), logistic (structuring a home care system is costly for municipalities) or even due to patients’ degree of dependence (i.e., those requiring noninvasive ventilation).^c^ In the case of patients requiring noninvasive ventilation, *Serviço de Apoio Domiciliar* has proven ineffective for lack of continuous structure (24h a day).^18^

Although it is clear that patients requiring tertiary emergency care have to be transferred to the hub municipalities, returning to their municipalities of origin is often hampered by the functional sequela (high dependency) that the emergency situation has brought about. In addition, it is accepted that the hub municipality should receive patients from other municipalities, but the smaller ones are unaware that when they receive patients from the hub municipality for high dependency care they benefit the whole system by increasing the supply of beds for tertiary emergencies.^1^ These intermunicipal health partnerships could contribute significantly, which is even more pertinent in light of the policy of *Redes Regionais de Assistência à Saúde* (Regional Networks of Health Care) of the Brazilian Ministry of Health, which includes the special subsidy for patients in need of long stays.^13^ ^,^ ^d^ The feasibility of this strategy was shown for the first time by this study.

Various efforts have been made for the high dependency nursing classification. Although they are contributing greatly to determine the staff necessary to provide this care, they have not progressed in the design of strategies for relocating these patients in institutions of lower complexity, optimizing SUS tertiary resources.^3^ ^,^ ^8^ ^,^ ^11^ ^,^ ^17^ Establishing patient dependency classifications could impact on referral management. However, qualifying health professionals in specific skills proved to be the most important action, including not only nursing professionals, but the entire multidisciplinary health care team. After qualification, health professionals in the partner hospitals started feeling more confident about patient care and demanding better working conditions. In addition, the Charlson index proved to predict death and return to the hospital of origin, being easily estimated using administrative data. This article reinforces the prognostic character of this index and its inability to predict patients’ hospital stay.^7^

We highlight the difficulty found in convincing patients and family of the benefits of the transfer for the patient. Many cases were not transferred due to family refusal, fearing that going away from the tertiary institution would impair their treatment. To convince them, we invited family members to visit the partner hospitals and obtained resources as the guarantee of direct return to the tertiary hospital if necessary, without intervention of the Regulation, and transportation assistance to visit patients.

Family members reluctant to the transfer were stimulated to think of alternative care strategies in other health spaces, including at home. Introducing this project has also made the multidisciplinary team aware of the daily need for case management and planning, in order to reduce the length of stay in the tertiary unit and expand the use of available resources in patients’ municipalities of origin, enabling greater case-resolving capacity and safe discharges.

The impact of long-stay beds on the system depends on the quantity installed, because the increase in common beds was only observed when their number went from 15 to 30. The necessary number is still unknown; however, the transfer capability of the tertiary hospital will probably be limited in more complex cases that require an intermediate institution between the tertiary and partner hospitals. Furthermore, continuing qualification can improve transfer capability. Regardless of the full training provided for in the ordinance, we observed 33.3% patient discharges.

The increase in discharges from the tertiary institution in stage 2 of the project did not result in a lower number of hospitalizations, as expected. This is probably due to pent-up demand from the DRS XIII, which started having more access to the tertiary hospital.^16^

This study also showed high mortality (41.7%), which is unsurprising, considering the degree of comorbidities and the high dependence of referred patients. The deaths occurred predominantly in patients with cerebrovascular diseases, specifically those with severe stroke sequelae. Time to death was similar among the hospitals observed, indicating that appropriate care was provided, since a shorter time would be expected in hospitals providing care inconsistent with patient needs. The greatest mortality observed in Guariba can be explained by the increased referral of patients in serious condition. Part of the purpose of the partner hospitals is the palliative care of patients with guarded prognoses. These institutions should also be qualified for that.

Besides the increased qualification of partner hospitals teams, the strategy presented another qualitative benefit: biweekly or monthly interaction between the various bodies that discuss the difficulties encountered and propose solutions. For instance, an outpatient clinic was structured specifically for patients discharged from partner municipalities, with the aim of all patients being seen in up to 15 days after discharge. According to data from the literature, this is the critical period of greater chance of being hospitalized again for lack of continued care.^15^

For the extension of this effort, it is necessary to strengthen medical transportation between institutions. Currently few transfers are made (about eight per month) and they are absorbed by the regional SAMU. Yet, with the increase in the number of beds to 120 and of institutions patients may be referred to, SAMU will probably be unable to cope with this demand. It is necessary to discuss a strategy to use another service for these referrals.

The data presented must be generalized carefully, because the benefit depends on the installed infrastructure. Areas with fewer resources that the DRS XIII of the state of Sao Paulo may not benefit in the same magnitude and the proposed incentive may be insufficient.^9^ ^,^ ^21^

In conclusion, implementing long-stay beds increases significantly the number of available beds in tertiary care, both in general and in system bottleneck areas such as Neurology and Intensive Care. The Charlson index of comorbidity is associated with the chance of patient death or return to tertiary care, even when adjusted for possible confounding factors.

## Footnotes

a Ministério da Saúde. Portaria MS nº 2.809, de 7 de dezembro de 2012. Estabelece a organização dos Cuidados Prolongados para retaguarda à Rede de Atenção às Urgências e Emergências (RUE) e às demais Redes Temáticas de Atenção à Saúde no âmbito do Sistema Único de Saúde (SUS). Brasília (DF): 2012 [cited 2015 Sept 24]. Available from: http://bvsms.saude.gov.br/bvs/saudelegis/gm/2012/prt2809_07_12_2012.html

b Considering that USD 1.00 was equivalent to BRL 2.40 in 2012, the incentive amounted to USD 125.00. The regular SUS *per diem* rate is BRL 30.00, i.e., USD 12.50.

c Ministério da Saúde. Portaria n^o^ 963, de 27 de maio de 2013. Redefine a Atenção Domiciliar no âmbito do Sistema Único de Saúde (SUS). Brasília (DF); 2013 [cited 2015 Sept 24]. Available from: http://bvsms.saude.gov.br/bvs/saudelegis/gm/2013/prt0963_27_05_2013.html

d Ministério da Saúde. Portaria nº 4.279, de 30 de dezembro de 2010. Estabelece diretrizes para a Organização das Redes de Atenção à Saúde no âmbito de Sistema Único de Saúde (SUS). Brasília (DF); 2010 [cited 2015 Sept 24]. Available from: http://bvsms.saude.gov.br/bvs/saudelegis/gm/2010/prt4279_30_12_2010.html
