# Supplementary material for: Impact of long-stay beds on the performance of a tertiary hospital in emergencies
Source: Rev Saude Publica. 2015 Nov 16;49:83. doi: 10.1590/S0034-8910.2015049006078 (PMC4650935; doi:10.1590/S0034-8910.2015049006078)
Supplement: Translation

# Translation: Impacto de leitos de longa permanência no desempenho de hospital terciário em emergências

**Authors:** Antonio Pazin, Edna de Almeida, Leni Peres Cirilo, Frederica Montanari Lourençato, Lisandra Maria Baptista, José Paulo Pintyá, Ronaldo Dias Capeli, Sonia Maria Pirani Felix da Silva, Claudia Maria Wolf, Marcelo Marcos Dinardi, Sandro Scarpelini, Maria Cecília Damasceno

**Affiliations:** I Divisão de Emergências Clínicas. Departamento de Clínica Médica. Faculdade de Medicina de Ribeirão Preto. Universidade de São Paulo. Ribeirão Preto, SP, Brasil; II Unidade de Emergência. Hospital das Clínicas. Faculdade de Medicina de Ribeirão Preto. Universidade de São Paulo. Ribeirão Preto, SP, Brasil; III Departamento de Atenção à Saúde. Unidade de Emergência. Hospital das Clínicas. Faculdade de Medicina de Ribeirão Preto. Universidade de São Paulo. Ribeirão Preto, SP, Brasil; IV Departamento Regional de Saúde XIII. Secretaria de Saúde do Estado de São Paulo. Ribeirão Preto, SP, Brasil; V Divisão de Cirurgia do Trauma. Departamento de Cirurgia e Anatomia. Faculdade de Medicina de Ribeirão Preto. Universidade de São Paulo. Ribeirão Preto, SP, Brasil; VI Departamento Clínico-Cirúrgico. Faculdade de Medicina do ABC. São Paulo, SP, Brasil

**Keywords:** Ocupação de Leitos, Número de Leitos em Hospital, Tempo de Internação, Assistência de Longa Duração, Atenção Terciária à Saúde, Serviços Médicos de Emergência, Índice de Comorbidade de Charlson

## Abstract

**OBJETIVO:**

Avaliar o impacto da implantação de leitos de longa permanência para pacientes de baixa complexidade e alta dependência em hospitais de pequeno porte sobre o desempenho de hospital terciário de referência em emergência.

**MÉTODOS:**

Para este estudo longitudinal, foram identificados hospitais em três municípios no departamento regional de saúde coberto pela instância terciária e que forneciam 10 leitos de longa permanência cada. Os pacientes foram transferidos para os hospitais desses municípios com base em protocolo específico. Obteve-se o desfecho dos pacientes transferidos por acompanhamento diário. Fatores de confusão foram ajustados por regressão logística e semiparamétrica de Cox.

**RESULTADOS:**

Entre 1 de setembro de 2013 e 30 de setembro de 2014, foram transferidos 97 pacientes, sendo 72,1% homens, com idade média de 60,5 anos (DP = 1,9), para os quais foram realizadas 108 transferências. Desses pacientes, 41,7% evoluíram ao óbito, 33,3% receberam alta, 15,7% retornaram à instância terciária, e apenas 9,3% permaneceram internados até o final do período analisado. Foi calculado o índice de comorbidade de Charlson – 0 (n = 28 [25,9%]), 1 (n = 31 [56,5%]) e ≥ 2 (n = 19 [17,5%]) – a única variável que aumentou a chance de óbito ou retorno ao hospital terciário (Razão de Chances = 2,4; IC95% 1,3;4,4). O tempo de permanência nos leitos de longa permanência foi de 4.253 pacientes-dia, que representariam 607 vagas no hospital terciário, considerando-se a média de internação de sete dias. O hospital terciário aumentou o número de vagas em 50,0% para terapia intensiva, 66,0% para neurologia e 9,3% para as vagas totais. A permanência dos pacientes nos leitos de longa permanência limitou-se em grande parte aos primeiros 30 (50,0%) e 60 (75,0%) dias.

**CONCLUSÕES:**

A implantação de leitos de longa permanência teve impacto no aumento de vagas novas oferecidas pela instância terciária tanto gerais como para áreas de estrangulamento do sistema, como a Neurologia e Terapia Intensiva. O índice de comorbidade de Charlson está associado à chance de o paciente evoluir ao óbito ou retornar para a instância terciária, mesmo quando ajustado por possíveis fatores de confusão.

## INTRODUÇÃO

Um dos maiores problemas do Sistema Único de Saúde (SUS) é a transferência de pacientes crônicos com alta dependência (necessidades de auxílio para funções vitais básicas), mas que deixaram de ser de alta complexidade (já não requerem cuidado médico ou recursos diagnósticos ou terapêuticos de nível terciário).^4^ ^,^ ^7^ Isso implica em permanência prolongada desses pacientes em sala de urgência e leitos hospitalares de internação geral em nível terciário, gerando atritos internos e externos,^19^ ^,^ ^20^ sobrecarga de trabalho para a equipe de enfermagem e elevados custos hospitalares.^3^ ^,^ ^8^ ^,^ ^10^

Para resolver esse problema, busca-se a instalação de leitos de longa permanência.^5^ Esses leitos estão previstos para serem alocados em hospitais de pequeno porte, que enfrentam enormes dificuldades econômicas para subsistência e têm baixa taxa de ocupação.^10^ Além disso, essas instituições têm dificuldades em lidar com pacientes de alta dependência com características especiais, como os dependentes de ventilação não-invasiva. O despreparo dessas instituições para lidar com a alta dependência causa alto índice de agravos agudos de condições crônicas, com elevadas taxas de contra referência para salas de urgência.^1^ ^,^ ^2^ ^,^ ^16^ Para que essa instalação seja possível, é previsto pagamento diferenciado para a diária e auxílio inicial de custo para qualificação.^a^

O objetivo deste estudo foi avaliar o impacto da implantação de leitos de longa permanência para pacientes de baixa complexidade e alta dependência em hospitais de pequeno porte sobre o desempenho de hospital terciário de referência em emergência.

## MÉTODOS

O estudo envolveu o hospital terciário de emergências e hospitais parceiros do DRS XIII. A Unidade de Emergência do Hospital das Clínicas da Faculdade de Medicina de Ribeirão Preto da Universidade de São Paulo (UE-HCFMRP-USP) dispõe de 169 leitos (30,0% de terapia intensiva), com sala de urgência referenciada, que serve de referência terciária para emergências do DRS XIII e quatro outros departamentos. Dispõe de recursos diagnósticos e terapêuticos de alta complexidade e se constitui na única referência para algumas condições clínicas num raio de 300 quilômetros no nordeste do estado de São Paulo.^1^ ^,^ ^14^

A Coordenação da Unidade de Emergência, o DRS XIII, a Prefeitura de Ribeirão Preto e o Serviço de Atendimento Móvel de Urgência (SAMU) Regional identificaram três hospitais para estabelecer parcerias localizados nos municípios de São Simão, Guariba e Altinópolis (Figura 1). Foram realizadas visitas técnicas *in loco* e estabelecidos protocolos de encaminhamento de pacientes de longa permanência, documentando as capacidades e as responsabilidades de cada instituição.

**Figura 1:** Municípios do Departamento Regional de Saúde XIII do Estado de São Paulo classificados quanto ao distrito. A esfera maior marca o município-pólo e as esferas menores, os municípios parceiros do projeto, mostrando sua localização estratégica para cada distrito.

A seleção dos hospitais parceiros se embasou no perfil exigido pela Portaria 2.809, de 7 de dezembro de 2012 e pela disponibilidade em assumir o risco de realizar o projeto com recursos próprios até o recebimento dos recursos oferecidos pelo estado de São Paulo. O envolvimento no projeto não ocorreu de modo simultâneo, haja vista que o preparo para receber pacientes foi variável, sendo que o primeiro se inseriu cerca de três meses antes do último. Esses hospitais tinham condições iniciais diferentes para receber pacientes, sendo que um deles já dispunha de preparo para pacientes de maior gravidade, enquanto os demais tiveram que ser capacitados. Também foi considerada a posição estratégica desses municípios (Figura 1).

Firmou-se um acordo entre os participantes para que o processo fosse benéfico para todas as partes (processo “ganha-ganha”). Para o hospital terciário, haveria o ganho de liberação de vagas para receber mais pacientes de alta complexidade. Para os hospitais-parceiros, haveria a perspectiva de ganho financeiro, pois para cada paciente-dia seria fornecido um incentivo de R$300,00 (trezentos reais).^b^ Além disso, esses hospitais estariam conectados ao hospital terciário e receberiam capacitação de seus funcionários.

Após visitas *in loco*, foi estabelecido protocolo de transferência com base nas condições físicas e de pessoal dos parceiros. O processo de transferência se iniciava com a concordância do paciente e seus familiares, sendo provida visita presencial de um familiar ao hospital-parceiro para avaliar as condições e a garantia de fornecimento de auxílio-transporte pelas prefeituras dos municípios do qual o paciente era residente para que os familiares continuassem a visitar o paciente após a transferência. Foi esclarecido que, se houvesse necessidade de retorno do paciente ao hospital terciário, isso seria feito independentemente da Regulação Médica.

Após a concordância do paciente e seus familiares, foi feita solicitação de vaga para o município-parceiro, escolhido de acordo com a proximidade do município de residência do paciente, pela disponibilidade de vagas ou por condições específicas do hospital de acordo com o problema apresentado pelo paciente. Nenhum paciente foi transferido sem o acordo mútuo das equipes de saúde das instituições (terciária e parceira) e do paciente ou seu familiar. Se o município parceiro concordasse com a transferência, o paciente era encaminhado com relatório médico, de enfermagem, nutricional, fisioterapêutico, psicológico e de assistência social. Esses pareceres detalhavam as necessidades dos pacientes para que o tratamento pudesse ser continuado no hospital parceiro.

Inicialmente, foram estabelecidos cinco leitos em cada hospital parceiro. Como nessa fase (de setembro de 2013 a março de 2014) ainda não havia recursos financeiros disponíveis, cada entidade envolvida no projeto contribuiu de alguma forma, com fornecimento de fraudas geriátricas ou medicamentos, por exemplo. O SAMU regional realizou as transferências em horários comerciais de segunda a sexta e todas as transferências foram programadas. Os funcionários dos hospitais parceiros foram treinados, pelo hospital de referência, quanto aos cuidados que limitassem as transferências, como o manejo de traqueostomia, uso de Bipap e preparo de dietas especiais.

A partir de abril de 2014, o projeto foi sancionado e apoiado financeiramente pela Secretaria da Saúde do Estado de São Paulo. Nessa fase, o número de leitos em cada parceiro foi elevado para 10, totalizando 30 leitos de longa permanência para o DRS XIII e outros hospitais do município-polo passaram a transferir pacientes seguindo as diretrizes criadas. O pagamento era feito pelo DRS XIII com recursos da Secretaria do Estado de São Paulo, mediante a produção apresentada. Se um parceiro mantivesse seus 10 leitos ocupados durante todo o mês, seu faturamento com esses leitos corresponderia a três a quatro vezes o seu faturamento total mensal.

O hospital terciário e o DRS XIII mantiveram controle de todos os pacientes internados nos parceiros diariamente. O paciente só deixaria de ser acompanhado quando deixasse o hospital parceiro (por óbito ou alta), tornando esses hospitais parceiros uma extensão da unidade terciária de origem.

As variáveis categóricas foram expressas em percentagem e as variáveis quantitativas, em média e desvio padrão ou mediana e intervalo interquartil, conforme distribuição. Para a comparação das variáveis categóricas, utilizou-se o teste de Fisher e o teste Qui-quadrado. Para a comparação das variáveis contínuas foi utilizado o teste *t* de Student paramétrico e análise de variância (ANOVA) ou seus equivalentes não-paramétricos. Utilizou-se análise de sobrevida para avaliar o tempo de permanência dos pacientes encaminhados em cada hospital parceiro. Nesses casos, alta, óbito ou transferência foram considerados desfechos.

Para a análise multivariada, utilizou-se a regressão logística, considerando-se como desfechos óbito ou transferência e regressão semi-paramétrica de Cox. Utilizou-se como desfecho o tempo efetivo que o paciente permaneceu nos parceiros (censurando em alta, óbito, transferência ou estar internado até 30 de setembro de 2014). Foi realizada análise de sensibilidade para avaliar exclusivamente o óbito como desfecho. Para ambas as estratégias, construiu-se modelos incrementais (*forward*) até o modelo final ajustado para idade, gênero, município parceiro e índice de Charlson. Foi calculado o índice de comorbidade de Charlson com base no código internacional de doenças CID-10.^12^ Para todos os testes, considerou-se significância estatística valores de p < 0,05. Para análise dos dados e construção dos gráficos, utilizou-se o programa *Stata* versão 10, Microsoft Excel^®^ e ArcGIS versão 9.

O projeto foi aprovado no Comitê de Ética em Pesquisa do Hospital das Clínicas da Faculdade de Medicina de Ribeirão Preto da Universidade de São Paulo (CAE 30686214.2.0000.5440). Foi obtida a dispensa do termo de consentimento livre e esclarecido para os pacientes por se tratar de estudo observacional envolvendo dados administrativos.

## RESULTADOS

Entre 1 de setembro de 2013 e 30 de setembro de 2014, foram incluídos 97 pacientes (72,1% do sexo masculino) com idade média de 60,5 (DP = 1,9) anos para os quais foram realizadas 108 transferências. A Tabela 1 descreve a distribuição de acordo com o hospital-parceiro, clínica de origem do paciente e evolução na instituição parceira. As transferências ocorreram principalmente em pacientes com problemas cardiovasculares e doenças degenerativas (Grupo Clínico), acidente vascular encefálico e outras afecções neurológicas (Grupo Neurológico) e trauma (Grupo Cirúrgico), com o intuito de complementação do tratamento (Tabela 1).

**Tabela 1:** Distribuição das transferências realizadas de acordo com o município, tipo de clínica de origem e desfecho no hospital de longa permanência.

	Alta	Óbito	Internado	Retorno	Total	%
Altinópolis (25,9%)						
Grupo Clínico	3	2	1	0	6	5,6
Grupo Neurológico	6	4	0	3	13	12,0
Grupo Cirúrgico	5	3	0	1	9	8,3
Guariba (56,5%)						
Grupo Clínico	3	9	1	4	17	15,7
Grupo Neurológico	6	14	2	3	25	23,1
Cirurgia	11	4	2	2	19	17,6
São Simão (17,6%)						
Grupo Clínico	1	1	1	1	4	3,7
Grupo Neurológico	0	7	2	2	11	10,2
Grupo Cirúrgico	1	1	1	1	4	3,7
Total	36	45	10	17	108	
%	33,3	41,7	9,3	15,7		

Nota: As transferências neurológicas corresponderam a 45,4% das transferências, enquanto aquelas por motivo clínico e cirúrgico corresponderam a 25,0% e 29,6%, respectivamente.

Foi calculado o índice de comorbidade de Charlson para os pacientes encaminhados para os hospitais parceiros (Tabela 2). Com relação ao desfecho, observou-se índice ≥ 2 em 5,0% dos que tiveram alta, 20,0% dos internados, 35,0% dos que tiveram que retornar ao serviço de origem e 40,0% dos que foram à óbito.

**Tabela 2:** Índice de Charlson de acordo com o município-parceiro.*

	0		1		≥ 2		Total	
Parceiro	n	%	n	%	n	%	n	%
Altinópolis	11	23,4	12	36,3	5	17,8	28	25,9
Guariba	30	63,8	17	51,5	14	50,0	61	50,5
São Simão	6	12,7	4	12,1	9	32,1	19	17,6
Total	47		33		28		108	100

* As porcentagens correspondem às colunas da tabela.

A Figura 2 ilustra o impacto dos leitos de longa permanência sobre o número de leitos novos oferecidos por mês para a Terapia Intensiva e para a Neurologia. Com relação aos leitos em geral, esses e as demais clínicas beneficiadas totalizaram 9,3% de aumento nos leitos novos oferecidos. Esse número total de altas foi observado após acréscimo de 15 para 30 leitos de longa permanência.

**Figura 2:** Número de vagas novas oferecidas para Terapia Intensiva e Neurologia (Linhas - Eixo Y à esquerda) pela instituição terciária de acordo com o número de pacientes transferidos para hospitais de longa permanência (Colunas - Eixo Y à direita) em função do tempo (Eixo X). A seta não preenchida aponta o início da Fase 1 do projeto e a seta preenchida aponta o início da Fase 2.

O cálculo da permanência total no hospital terciário e nos leitos de longa permanência foi de 9.134 pacientes-dia. Desses, 4.881 foram do hospital terciário (53,5%) e 4.253 dos leitos de longa permanência (46,5%). Considerando-se o tempo médio de internação de sete dias no hospital-terciário, o tempo de permanência nos hospitais parceiros permitiria que aproximadamente 607 pacientes novos fossem atendidos.

Não houve diferença significativa entre os hospitais parceiros, tanto na análise univariada, quanto na multivariada pela regressão logística ou pela regressão semi-paramétrica de Cox (Figura 3). Cerca de 50,0% dos pacientes tiveram alta, evoluíram ao óbito ou foram transferidos nos primeiros 30 dias. O índice de Charlson foi a única variável que aumentou significantemente a chance de óbito ou retorno ao hospital terciário (Razão de Chances – 2,4; IC95% 1,3;4,4), mas não houve significância quanto ao tempo de permanência na regressão de Cox (Razão de Chances – 1,2; IC95% 0,8;1,7) (Figura 3).

**Figura 3:** Gráfico de Kaplan-Meier para o tempo de permanência de pacientes em cada instituição.

Os municípios foram semelhantes (p > 0,05) quanto ao percentual de óbitos – Altinópolis (9; 32,1%), Guariba (27; 44,2%) e São Simão (9; 47,3%). Os pacientes que evoluíram ao óbito foram mais frequentes no Grupo Neurológico (25; 51%), seguidos do Grupo Clínico (12; 44,4%) e do Grupo Cirúrgico (8; 25,0%). Segundo os códigos da CID-10, as principais causas de óbito foram: Doenças Circulatórias (20; 45,5%), Trauma (7; 15,9%), Doenças do Aparelho Respiratório (7; 15,9%), Neoplasias (4; 9,1%) e Outros (6; 13,6%). Ao se examinar os códigos CID-10 mais frequentes, observou-se que os decorrentes de doenças cerebrovasculares responderam por 29,5% dos casos, dos quais 60,0% foram internados em Guariba. Quando se considerou apenas o óbito como desfecho na análise de regressão de Cox, nenhuma das variáveis incluídas no modelo foi significativa. Na análise de regressão logística multivariada, o índice de Charlson permaneceu como uma variável significante – OR = 1,7 (1,0;2,9) – e observou-se maior chance de óbito nos pacientes encaminhados para Guariba – OR = 1,8 (1,0;2,9).

## DISCUSSÃO

A introdução de leitos de longa permanência no DRS XIII teve impacto considerável no aumento de vagas oferecidas pela instância terciária para áreas de estrangulamento do sistema, como a Neurologia e Terapia Intensiva. Após a ampliação do projeto para 30 leitos, notou-se tendência de aumento de vagas gerais de atendimento no hospital terciário. O grau de comorbidade, mensurado pelo índice de Charlson, esteve associado à chance de o paciente evoluir ao óbito ou retornar para a instância terciária, mesmo quando ajustado por possíveis fatores de confusão. A permanência dos pacientes nos hospitais parceiros limitou-se, em grande parte, aos primeiros 30-60 dias.

A gestão plena da saúde pelos municípios tem gerado a compreensão de que cada município poderia ser responsável em fornecer esse apoio para pacientes de longa permanência, transferindo-os para instâncias primárias do SUS localizadas nos municípios de residência do paciente. Essas transferências são desejáveis, principalmente pelo benefício de manter o suporte familiar. Entretanto, a maioria dos municípios utiliza o sistema de compra de serviços de instituições hospitalares públicas ou filantrópicas, i.e., muitos municípios contam com apenas uma instituição de apoio ou mesmo nenhuma, o que inviabiliza a transferência.

Uma possível estratégia seria a desospitalização desses pacientes para o domicílio, adicionando-se o suporte estratégico de equipes de Saúde da Família e do Serviço de Apoio Domiciliar. Embora essa estratégia seja convidativa, ela esbarra em diversos problemas de ordem social (o cuidador é obrigado a deixar a atividade produtiva, com repercussões financeiras para o domicílio), logística (a estruturação de um Sistema de Apoio Domiciliar é onerosa para os municípios) ou mesmo decorrentes do grau de dependência do paciente (como aqueles que requerem ventilação não-invasiva).^c^ Nos casos de pacientes que requerem ventilação não-invasiva, o Serviço de Apoio Domiciliar tem se mostrado ineficaz, por não dispor de estrutura continuada (24h por dia).^18^

Muito embora já se tenha claro que pacientes que necessitem de cuidados terciários de emergência tenham que ser transferidos para os para municípios polos, o retorno desses pacientes para os municípios de origem muitas vezes é dificultado pela sequela funcional (alta dependência) que a situação de emergência ocasionou. Além disso, aceita-se o fato de que o município-polo deva receber pacientes de outros municípios, mas os municípios de menor porte não tem clareza de que ao receber pacientes do município-polo para cuidado de alta dependência, estarão beneficiando todo o sistema ao aumentar a oferta de vagas para situações de emergência terciária.^1^ Essa alternativa, na forma de parcerias intermunicipais de saúde, poderia contribuir significativamente, o que se torna ainda mais pertinente frente à política de Redes Regionais de Assistência à Saúde do Ministério da Saúde, que contempla o subsídio diferenciado para pacientes com necessidade de longa permanência.^13^ ^,^ ^d^ A exequibilidade dessa estratégia foi mostrada de modo inédito pelo presente trabalho.

Vários esforços foram empreendidos para a classificação de alta dependência em enfermagem que, embora estejam contribuindo muito para o delineamento da capacidade de trabalho necessária para prestar esses cuidados, não progrediram no delineamento de estratégias para realocação desses pacientes para instâncias de menor complexidade, otimizando os recursos terciários do SUS.^3^ ^,^ ^8^ ^,^ ^11^ ^,^ ^17^ O estabelecimento de classificações de dependência dos pacientes poderia ter impacto na gestão dos encaminhamentos. No entanto, a capacitação dos profissionais de saúde em habilidades específicas mostrou ser o mais importante, incluindo não somente os profissionais de enfermagem, mas toda a equipe multidisciplinar de saúde. Após a capacitação, os profissionais de saúde dos hospitais parceiros passaram a se sentir mais confiantes quanto ao cuidado dos pacientes e a exigir melhores condições de trabalho. Além disso, o índice de Charlson se mostrou preditivo de óbito e retorno ao hospital de origem, sendo facilmente calculado a partir de dados administrativos. Este artigo reforça o caráter prognóstico desse índice e sua incapacidade de prever a permanência hospitalar do paciente.^7^

Destaca-se a dificuldade encontrada em convencer pacientes e familiares dos benefícios da transferência para o próprio paciente. Muitos casos não foram transferidos em virtude da negativa dos familiares, com receio de que a retirada do paciente da instituição terciária prejudicasse o seu tratamento. Para que houvesse o convencimento, os familiares eram convidados a visitar os hospitais parceiros e foram obtidos recursos como a garantia de retorno direto ao hospital terciário se fosse necessário, sem intervenção da Regulação, e auxílio-transporte para visita aos pacientes.

Os familiares que resistiram à transferência para os hospitais parceiros foram convidados a pensar em estratégias alternativas de cuidado, em outros espaços de saúde, incluindo o próprio domicílio. A introdução desse projeto também despertou na equipe multidisciplinar a necessidade diária de gestão dos casos e planejamento, visando diminuir o tempo de permanência na unidade terciária e ampliar o uso de recursos disponíveis nos municípios de origem dos pacientes possibilitando maior resolutividade e alta segura.

O impacto de leitos de longa permanência no sistema depende da quantidade instalada, pois o aumento dos leitos gerais só foi observado quando se elevou de 15 para 30 leitos. Desconhece-se ainda o número necessário, porém provavelmente a capacidade de transferência do hospital terciário venha a ser limitada em casos mais complexos que exigiriam uma instância intermediária entre os hospitais terciário e parceiros. Além disso, a capacitação continuada poderá melhorar a capacidade de transferência. Independentemente da capacitação plena prevista em portaria, observou-se 33,3% de altas hospitalares.

O aumento de altas da instituição terciária na fase 2 do projeto não implicou em redução do número de hospitalizações, como esperado. Isso provavelmente se deve à demanda reprimida do DRS XIII que passou a ter maior acesso ao hospital terciário.^16^

Este estudo também mostrou elevada mortalidade (41,7%). Esse dado não surpreende, considerando o grau de comorbidades e a alta dependência dos pacientes encaminhados. Os óbitos ocorreram predominantemente em pacientes com doenças cerebrovasculares, especificamente em pacientes com sequelas graves de acidente vascular encefálico. O tempo para o óbito não diferiu entre os hospitais observados, indicando que o cuidado esteja sendo adequado, visto que seria esperado tempo menor naqueles em que o cuidado não estivesse condizente com a necessidade do paciente. A maior mortalidade observada em Guariba pode ser explicada pelo maior encaminhamento de pacientes em condições graves. Parte da função desses hospitais parceiros é o cuidado paliativo dos pacientes com prognóstico reservado. Esses estabelecimentos deverão receber capacitação também nesse sentido.

Além do aumento da capacitação das equipes dos hospitais parceiros, a estratégia apresentou outro benefício qualitativo: a interação quinzenal ou mensal entre as diversas instâncias que discutem as dificuldades encontradas e propõem soluções. Como exemplo, foi estruturado um ambulatório específico para os pacientes egressos dos municípios parceiros, com o objetivo de que todos os pacientes sejam vistos em, no máximo, 15 dias após a alta. Segundo dados de literatura, esse é o período crítico em que há maior chance de rehospitalização, por falta dos cuidados continuados que devem ser fornecidos nesse período.^15^

Para que essa iniciativa se amplie, é necessário o fortalecimento do transporte sanitário entre instituições. Atualmente, as transferências são em pequeno número (cerca de oito por mês) e estão sendo absorvidas pelo SAMU regional. Entretanto, com a ampliação do número de leitos para 120 e o aumento das instituições de referências que poderão encaminhar pacientes, provavelmente o SAMU não poderá arcar com essa demanda. Faz-se necessário discutir uma estratégia para que esses encaminhamentos sejam realizados por outro serviço.

A generalização dos dados apresentados deve ser feita com cuidado, pois o benefício depende da infraestrutura instalada. Regiões com menores recursos que os presentes no DRS XIII do estado de São Paulo podem não se beneficiar na mesma magnitude e o incentivo proposto pode ser insuficiente.^9^ ^,^ ^21^

Concluindo, a implantação de leitos de longa permanência aumenta consideravelmente as vagas novas oferecidas pela instância terciária, tanto gerais como para áreas de estrangulamento do sistema, como a Neurologia e Terapia Intensiva. O índice de comorbidade de Charlson está associado à chance de o paciente evoluir ao óbito ou retornar para a instância terciária, mesmo quando ajustado por possíveis fatores de confusão.

## Footnotes

a Ministério da Saúde. Portaria MS nº 2.809, de 7 de dezembro de 2012. Estabelece a organização dos Cuidados Prolongados para retaguarda à Rede de Atenção às Urgências e Emergências (RUE) e às demais Redes Temáticas de Atenção à Saúde no âmbito do Sistema Único de Saúde (SUS). Brasília (DF): 2012 [citado 2015 set 24]. Disponível em: http://bvsms.saude.gov.br/bvs/saudelegis/gm/2012/prt2809_07_12_2012.html

b Considerando que US$1,00 equivalia a R$2,40 em 2012, o valor pago pela diária equivalia a US$125,00. Uma diária normal do Sistema Único de Saúde equivale a R$30,00, ou seja, US$12,50.

c Ministério da Saúde. Portaria n^o^ 963, de 27 de maio de 2013. Redefine a Atenção Domiciliar no âmbito do Sistema Único de Saúde (SUS). Brasília (DF); 2013 [citado 2015 set 24]. Disponível em: http://bvsms.saude.gov.br/bvs/saudelegis/gm/2013/prt0963_27_05_2013.html

d Ministério da Saúde. Portaria nº 4.279, de 30 de dezembro de 2010. Estabelece diretrizes para a Organização das Redes de Atenção à Saúde no âmbito de Sistema Único de Saúde (SUS). Brasília (DF); 2010 [citado 2015 set 24]. Disponível em: http://bvsms.saude.gov.br/bvs/saudelegis/gm/2010/prt4279_30_12_2010.html
